# Influence of storage time on DNA of *Chlamydia trachomatis, Ureaplasma urealyticum*, and *Neisseria gonorrhoeae* for accurate detection by quantitative real-time polymerase chain reaction

**DOI:** 10.1590/1414-431X20165303

**Published:** 2016-08-25

**Authors:** Y. Lu, C.Z. Rong, J.Y. Zhao, X.J. Lao, L. Xie, S. Li, X. Qin

**Affiliations:** 1 Department of Clinical Laboratory, First Affiliated Hospital, Guangxi Medical University, Nanning, Guangxi, China; 2 Department of Clinical Laboratory, Children's Hospital, Maternal and Child Health Hospital, Guangxi Zhuang Autonomous Region, Nanning, Guangxi, China

**Keywords:** Storage time, Chlamydia trachomatis, Ureaplasma urealyticum, Neisseria gonorrhoeae, Stability, qRT-PCR

## Abstract

The shipment and storage conditions of clinical samples pose a major challenge to the detection accuracy of *Chlamydia trachomatis* (CT), *Neisseria gonorrhoeae* (NG), and *Ureaplasma urealyticum* (UU) when using quantitative real-time polymerase chain reaction (qRT-PCR). The aim of the present study was to explore the influence of storage time at 4°C on the DNA of these pathogens and its effect on their detection by qRT-PCR. CT, NG, and UU positive genital swabs from 70 patients were collected, and DNA of all samples were extracted and divided into eight aliquots. One aliquot was immediately analyzed with qRT-PCR to assess the initial pathogen load, whereas the remaining samples were stored at 4°C and analyzed after 1, 2, 3, 7, 14, 21, and 28 days. No significant differences in CT, NG, and UU DNA loads were observed between baseline (day 0) and the subsequent time points (days 1, 2, 3, 7, 14, 21, and 28) in any of the 70 samples. Although a slight increase in DNA levels was observed at day 28 compared to day 0, paired sample *t*-test results revealed no significant differences between the mean DNA levels at different time points following storage at 4°C (all P>0.05). Overall, the CT, UU, and NG DNA loads from all genital swab samples were stable at 4°C over a 28-day period.

## Introduction

*Chlamydia trachomatis* (CT) and *Neisseria gonorrhoeae* (NG) are the two most common bacteria responsible for sexually transmitted diseases (STD) worldwide, accounting for 131 million and 78 million of the total estimated 357 million new infections per year, respectively, according to the World Health Organization (http://www.who.int/mediacentre/factsheets/fs110/en/). *Ureaplasma* species, consisting of 14 serovars, which can be divided into two biovars – *U. parvum* and *U. urealyticum* (UU), are also frequently found in STD (1). However, *U. urealyticum* has been reported to be more pathogenic than *U. parvum* in several studies, and is the etiological agent in nongonococcal urethritis (NGU) (2). Other agents, such as the herpes simplex virus and Epstein Barr Virus, are found in a very small ratio, and their association with NGU is only suspected (3). Therefore, UU, CT and NG have been considered the major causes of urethritis and adverse pregnancy outcome, and are listed as routine test items in STD diagnosis ([Bibr B04] [Bibr B05] [Bibr B06]–7).

Traditionally, detection of CT, NG, and UU in clinical specimens relies on selective culture methods that are labor intensive, time consuming, and have a low sensitivity. The application of quantitative real-time polymerase chain reaction (qRT-PCR) has revolutionized pathogen diagnostics, having much higher sensitivities than the respective culture methods (95.7–98.7 *vs* 60–85%, 100 *vs* 86.8%, and 89.5 *vs* 47.4% in CT, NG, and UU detection, respectively) ([Bibr B08] [Bibr B09]–10). Furthermore, qRT-PCR can be performed on a variety of samples obtained through non-invasive methods (including urethral, urine, vaginal, and cervical specimens) (11), is rapid, and offers a high degree of automation. There are several commercially available RT-PCR systems allowing the analysis of 96–384 samples in 35–55 min (12). It has been previously reported that qRT-PCR enables a higher rate of UU detection compared to the culture method ([Bibr B13] [Bibr B14]–15), mainly due to a substantial improvement in the detection of very low levels of pathogens. In addition, it has been used to detect and distinguish the two biovars of UU (*U.urealyticum* and *U. parvum*) (16), which is far beyond the capabilities of the culture method.

Despite these advantages, not all hospitals are equipped or qualified to perform qRT-PCR, and therefore samples must often be transported to suitably equipped laboratories. In some parts of China, this would not normally present any major challenges; however, significant time delays may occur in some rural areas. Furthermore, clinical laboratories may be overloaded and hold an excessive number of specimens pending analysis or reevaluation. This raises concerns regarding the possible deterioration of DNA during storage. Since pre-analytical factors, including sample collection, processing, and storage conditions, may affect qRT-PCR detection, adverse storage conditions and duration may lead to an increased likelihood of false-negative results and misdiagnoses.

Unfortunately, few studies have addressed the influence of storage conditions on pathogen detection by qRT-PCR and the available results are mixed. For instance, van Dommelen et al. (17) demonstrated that storage conditions (room temperature, 4°C, -20°C, and -80°C) and duration of untreated specimens (after 0, 1, 7, 14, and 30 days, and 2 years of storage) had no significant effects on the detection of CT by qRT-PCR (136 clinical CT-positive urine and swab samples and 287 spiked samples). Conversely, Dize et al. (18) observed a moderate detection of CT in highly concentrated samples (10^4^ IFU/mL on dry swab) on day 90 and no detection in low-concentration swabs (10^3^ IFU/mL) after 40 days storage (all at 4°C). These contradictory results may be attributed to the type of swab used for sample collection, as some types of swabs have been shown to decrease the isolation rate or interfere with the qRT-PCR assay (11,19,20), or to the use of transport media compared to dry swabs. Indeed, transport media have been shown to affect pathogen storage duration (11,17,20). These results suggest that samples should be handled and stored according to their type; nevertheless, this is not always practically feasible. On the other hand, shipment of a relatively large volume of untreated clinical samples using cold storage packs greatly increases the costs of qRT-PCR testing in remote areas. If extracted DNA could be adequately stored and transported, inconvenience and costs would be greatly reduced. To date, few studies have assessed the storage duration of extracted DNA of CT, NG, and UU pathogens, particularly from clinical samples. Therefore, the present study assessed the impact of storage duration, for a period of up to 28 days, on the DNA of clinical CT-, NG-, or UU-positive specimens according to their detection using qRT-PCR.

## Material and Methods

### Ethical approval

Ethical approval was obtained from the Ethics Committee of the First Affiliated Hospital of Guangxi Medical University, Nanning, Guangxi, China.

### Study subjects

A total of 70 samples, collected from 70 patients who were initially screened as CT- (n=30), UU- (n=30), or NG-positive (n=10) at the Sexually Transmitted Diseases clinic of the First Affiliated Hospital of Guangxi Medical University, between June and September 2015, were included in the study. The study was fully explained to the patients and written informed consent was obtained prior to enrollment. The CT- and UU-positive samples were further divided into three groups according to their initial DNA loads: i) low concentration group, including 9 samples with low CT DNA loads and 10 samples with low UU DNA loads (mean: 3.34, range: 2.86–3.97 log_10_ copies/mL and mean: 3.47, range: 2.95–3.87 log_10_ copies/mL for CT and UU, respectively); ii) intermediate concentration group, including 11 samples with intermediate CT DNA loads and 10 samples with intermediate UU DNA loads (mean: 4.54, range: 4.06–4.93 log_10_ copies/mL and mean: 4.56, range: 4.15–4.92 log_10_ copies/mL for CT and UU, respectively), and iii) high concentration group, including 10 samples with high CT or UU DNA loads (mean: 6.53, range: 5.45–7.78 log_10_ copies/mL and mean: 5.53, range: 5.11–6.36 log_10_ copies/mL for CT and UU, respectively). Considering that once NG DNA is detected a patient is regarded as being NG-positive, only one 10-sample group was included in the NG DNA assays, comprising low to high DNA loads (3.94–7.75, mean: 6.29 log_10_ copies/mL). The basic characteristics of the study population are presented in Table 1.

**Table:**
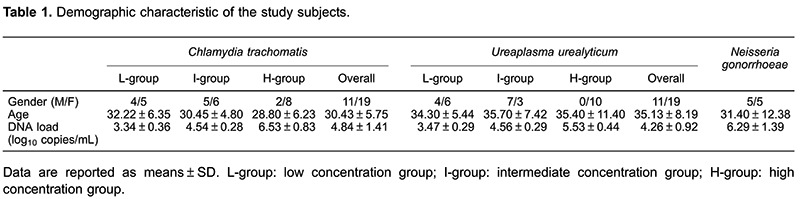

### Genital swab samples

Genital swabs (made of medical absorbent cotton, urethral swabs from males and vaginal swabs from females) were collected from all participants and placed into separate transport tubes (Yangzhou JiKang Medical Instrument Ltd., China) and were immediately transported to the Department of Clinical Laboratory in a dry tube environment, where they were hydrated and tested for CT, UU, and NG by qRT-PCR. Nucleic acid extraction and qRT-PCR were performed using CT, UU, and NG DNA Fluorescence Diagnostic Kits (Sansure Biotech, China; Product No. 3400143, 3400146, and 3400142, respectively, with Registration License (2011) by China Food, Drug and Medical Device Administration). DNA and nucleic acid extraction of the samples were performed following the manufacturer's recommended protocol. Briefly, the genital swab was rehydrated with 1 mL 0.9% normal saline and swirled vigorously for 1 min; then, 500 μL of the rehydrated sample were transferred to a 1.5-mL reaction tube (DNA LoBind Tubes, Eppendorf, Germany) and centrifuged at 15,800 *g* for 5 min at 4^o^C, 50 μL DNA extraction buffer was added to each tube after the supernatant was removed. Samples were mixed thoroughly, spun down briefly, and heated at 95°C for 10 min. Finally, each sample was centrifuged at 15,800 *g* for 5 min at 4^o^C and divided into 5-μL aliquots (DNA LoBind Tubes, Eppendorf). One aliquot of each sample was used immediately for qRT-PCR analysis, and the remaining aliquots were stored at 4^o^C and processed on days 1, 2, 3, 7, 14, 21, or 28.

### qRT-PCR analysis

A 5-μL template DNA from CT, UU, or NG as prepared by the previously described DNA extraction method was subjected to the commercially available PCR kits mentioned above. Amplification was performed using a SLAN-96P Real-Time PCR System (Sansure Biotech) with the following protocol: an initial 50°C, 2-min step for uracil N-glycosylase enzyme reaction followed by a 94°C, 5-min Taq enzyme activation step, 45 cycles at 94°C for 15 s and 57°C for 30 s for denaturation, an annealing (with fluorescence monitoring) step, and an elongation phase. Considering the possibility of false-positive results and the presence of qRT-PCR inhibition, both a negative and a positive control were included in each run. Finally, CT, UU, and NG concentrations were determined based on a four-point standard curve generated by amplifying 1×10^3^, 1×10^4^, 1×10^5^, and 1×10^6^ copies each of CT, UU, or NG during each amplification. The detection limit of these PCR kits was 4×10^2^ copies/mL, and their inter- and intra-assay coefficients of variation were all within 10%. Each qRT-PCR analysis for CT, UU, and NG samples was carried out in the same run by one technician using the same batch kit.

### Statistical analyses

Before analyses, the data were log_10_ transformed. A paired-sample *t*-test was performed using SPSS version 16.0 (SPSS Inc., USA) to assess any significant differences in concentrations of CT, UU, and NG at different storage times. All data are reported as means±SD and the significance level was set at P<0.05.

## Results

**Figure 1:**
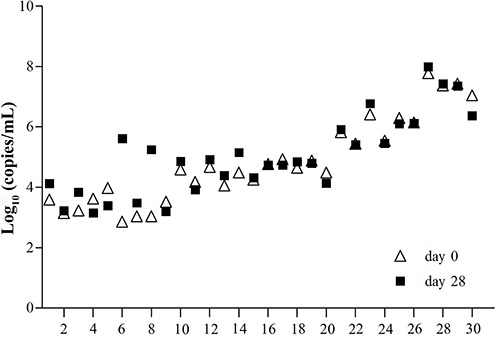
Comparison of *Chlamydia trachomatis* DNA concentrations using extracted DNA at day 0 and at day 28 of storage at 4°C.

CT, UU, and NG samples from the 70 patients were successfully analyzed, without any inhibition or false-positive results. The DNA concentrations at days 0, 1, 2, 3, 7, 14, 21, and 28 are shown in Table 2. For CT DNA detection, the mean level in the low-concentration group ranged from 3.34 to 3.92 log_10_ copies/mL. Interestingly, an increased DNA level was observed at day 28 compared to day 0, but this difference was not statistically significant (P=0.069). Data from all 30 samples tested in days 0 and 28 are also shown in Figure 1, which shows a near overlap for sample 2 between days 0 and 28. With regards to the intermediate concentration group, the mean concentration ranged from 4.51 to 4.85 log_10_ copies/mL, showing a similar non-significant increase between days 0 and 28 (P=0.677). Three overlaps (samples 15, 16, and 19) were found among the samples tested at days 0 and 28 in this group (Figure 1). The high-concentration group had a high CT DNA level in the range 6.53-6.69 log_10_ copies/mL, which decreased from 6.53 to 6.49 log_10_ copies/mL over the 28-day period. However, the differences were not statistically significant and half of the sample was overlapped between days 0 and 28.

**Table:**
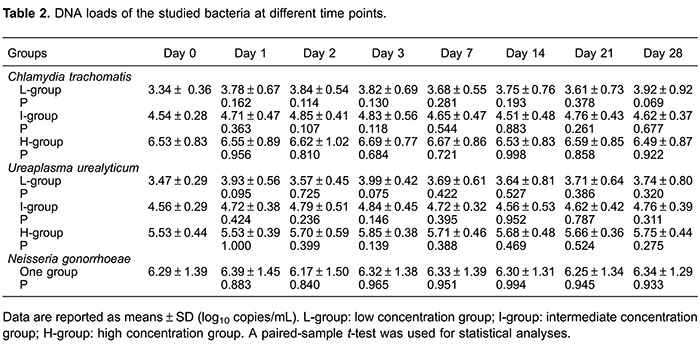

**Figure 2:**
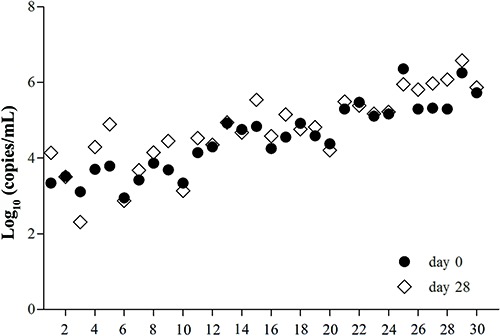
Comparison of *Ureaplasma urealyticum* DNA concentrations using extracted DNA at day 0 and at day 28 of storage at 4°C.

For the UU DNA analyses, the concentration of the low UU DNA group ranged from 3.47 to 3.99 log_10_ copies/mL, increasing from 3.47 to 3.74 log_10_ copies/mL over the 28-day period, although the difference was not significant; two overlaps (samples 2 and 6) were observed (Figure 2). The intermediate and high UU DNA concentration samples showed similar results, with DNA levels ranging from 4.56 to 4.84 and 5.53 to 5.85 log_10_ copies/mL, respectively, and with no differences being statistically significant (Table 2).

**Figure 3:**
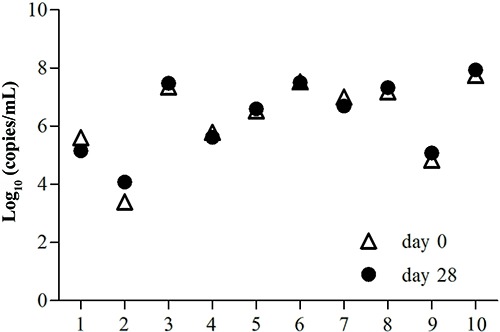
Comparison of *Neisseria gonorrhoeae* DNA concentrations using extracted DNA at day 0 and at day 28 of storage at 4°C.

The mean NG DNA concentration ranged from 6.17 to 6.39 log_10_ copies/mL. During the 28-day period, the mean NG DNA increased from 6.29 to 6.34 log_10_ copies/mL, but similar to the previous results, the difference was not statistically significant (P=0.933). Three overlaps between samples tested at days 0 and 28 were observed (samples 4, 5, and 6; Figure 3).

## Discussion

Given the rapid, specific, sensitive, and quantitative detection of CT, NG, and UU DNA, qRT-PCR is becoming the method of choice in research and screening programs with a centralized batch processing of clinical specimens (19). However, hospitals in remote areas and with limited laboratory capacities still have to transport samples to distant clinical laboratories that offer DNA testing. Thus, optimized sample collection, transportation, and storage of the specimens are of great importance. The detection ability of untreated specimens of CT, NG, and UU following various storage conditions using qRT-PCR has been explored in several studies (17,18,21), albeit with controversial results. The storage of extracted DNA should be practical both for sample transportation and further sample reanalysis. Nevertheless, the effect of different storage conditions on the detection of the extracted DNA load of these organisms using qRT-PCR has not been thoroughly assessed in clinical specimens. The present study therefore assessed the optimum DNA storage conditions to ensure the accuracy and reproducibility of test results with qRT-PCR.

The results presented herein show that no significant differences in CT, NG, and UU DNA loads were observed between immediate detection and detection following storage, in any of the 70 samples. In addition, for CT and UU detection, the collected samples were further divided into three categories based on their range of DNA concentration, including samples with low, intermediate, and high CT or UU DNA loads. Importantly, similar non-significant results were observed after stratification. These findings indicate that the DNA of CT, NG, and UU stored at 4°C is stable for at least 28 days, regardless of the initial DNA concentration. Thus, if samples cannot be immediately assayed, their DNA could be extracted and stored at 4°C for up to 28 days, and subsequently transported if necessary.

Freise et al. (22) explored the influence of DNA storage on the sensitivity of detection limits of CT elementary bodies (EB) at −20°C for up to 4 months; however, dramatically decreased detection limits (10- to 1000-fold) for CT EBs were observed. These results are inconsistent with those presented herein, and may be attributed to the following observations: first, the different DNA extraction methods used could lead to varying detection sensitivities after DNA storage. As reported by Freise et al. (22), an average 1000-fold decrease in detection rate was observed when DNA was extracted using Qiaex II Gel Extraction Kit¯+CTAB, while a 10-fold decrease was found when using alkaline lysis. Secondly, only one time-point (4 months) was used in their study. Finally, samples with serial dilutions of cultured CT EB were used in their study, which may not reflect real clinical samples. Therefore, our results are not comparable to those of that study, given the different DNA extraction methods, storage temperature, and time-points used. Given that few studies have assessed the impact of storage conditions on the accuracy of qRT-PCR DNA detection, further studies with larger numbers of clinical specimens should be conducted to confirm these results as well as to determine whether sample stability would be observed for CT, NG, and UU DNA loads after much longer term storage.

Overall, the CT, UU, and NG DNA loads from all genital swab samples were stable at 4°C over a 28-day period. These findings can therefore be applied in the shipment of clinical specimens, when analysis is postponed due to a laboratory backlog, or when samples require reevaluation to confirm prior results.
